# Neuromuscular fatigue and loss of torque complexity during severe‐intensity exercise: The role of muscle size

**DOI:** 10.1113/EP092972

**Published:** 2026-04-01

**Authors:** Rubens Correa Junior, Renan Vieira Barreto, Gabriel Fontanetti, Leonardo Coelho Rabello de Lima, Benedito Sérgio Denadai, Camila Coelho Greco

**Affiliations:** ^1^ Human Performance Laboratory, Department of Physical Education São Paulo State University Rio Claro São Paulo Brazil; ^2^ MioEx, School of Physical Education and Sport of Ribeirão Preto University of São Paulo Ribeirão Preto São Paulo Brazil

**Keywords:** critical torque, exercise tolerance, handgrip, knee extensor, performance

## Abstract

This study investigated the effect of severe‐intensity exercise on neuromuscular fatigue (NMF) and torque complexity in two muscle groups of different sizes. The NMF of 11 healthy males was assessed through maximal voluntary contractions at the beginning, every minute and at task failure during intermittent isometric exercise performed at an intensity 15% above the critical torque for knee extensors (KE) and handgrip (HG) muscles. Torque complexity was assessed using submaximal contractions from the first and last minutes of the exercise through approximate entropy (ApEn) and detrended fluctuation analysis (DFA‐α). The NMF reduced torque complexity (ApEn from 1.55 ± 0.09 to 1.25 ± 0.20, *P* < 0.001; DFA‐α from 1.40 ± 0.11 to 1.52 ± 0.06, *P* = 0.001) of the KE muscles. However, in HG muscles, the torque complexity was reduced only for DFA‐α analysis (1.01 ± 0.16 to 1.24 ± 0.16, *P* < 0.001). The loss of complexity was accompanied by greater central (KE −14.9 ± 12.4% × HG −7.66 ± 5.23%, *P* < 0.001) and peripheral fatigue (KE −63.8 ± 20.9% × HG −29.0 ± 14.5%, *P* < 0.001) in KE muscles than HG muscles, despite a similar decline in maximal torque production and exercise tolerance. These findings suggest that the magnitude of NMF may influence the loss of torque complexity between different muscle groups, resulting in impaired motor control ability. Furthermore, the greater impairment observed may be influenced by differences in muscle size, which could affect various physiological responses.

## Abstract (highlights)

- **What is the central question of this study?**
  Do differences in muscle size influence the loss of torque complexity during a task performed to failure within the severe‐intensity domain?
- **What is the main finding and its importance?**
  The loss of torque complexity was more pronounced in the muscle experiencing greater central and peripheral fatigue, which led to greater fluctuations in torque output, and consequently, a decreased motor control ability, despite a similar decline in maximal torque production and tolerance to severe‐intensity exercise.

## 1 INTRODUCTION

In both non‐fatigued and fatigued muscles, torque output patterns exhibit fluctuations over time, which can be quantified in terms of their complexity (e.g. approximate entropy, ApEn, and detrended fluctuation analysis, DFA‐α) (Pethick et al., 2021). ApEn quantifies the regularity or randomness of torque fluctuations, whereas DFA‐α demonstrates their temporal fractal scaling (Pincus, 1991; Slifkin & Newell, 1999). The complexity of torque output is a measure of the neuromuscular system's adaptability to internal or external perturbations. When the neuromuscular system is under stress due to fatigue, adaptability is impaired, thereby leading to higher torque unsteadiness and reduced capacity to modulate torque accurately (Pethick et al., 2021).

Neuromuscular fatigue (NMF) induces a reversible decline in torque production because of peripheral and central failures (Allen et al., 2008; Gandevia, 2001), simultaneously reducing torque complexity, as shown in previous studies (Pethick et al., 2015, 2016; Penthick, Winter et al., 2019). This loss of complexity impairs motor control and negatively affects exercise performance (Pethick, Winter et al., 2019). In addition, recent studies have revealed that the extent of loss in torque complexity is intensity‐dependent, with both maximal and submaximal exercises leading to significant reductions (Pethick et al., 2015; Penthick, Winter et al., 2019). However, Pethick et al. (2016) observed that during submaximal contractions, loss of torque complexity occurred only for exercise intensities that exceeded critical torque (CT), demonstrating that this decline occurs only in the severe‐intensity domain, where NMF develops progressively to the point of task failure.

Although these recent findings highlight the significant impact of exercise intensity on the loss of torque complexity, several studies have investigated only a single muscle group. As a result, it is unclear whether different muscles show distinct responses when performing the same task, particularly when comparing muscles of different sizes and functional roles during daily life, such as the handgrip (HG) and knee extensors (KE) muscles. It is interesting to note that these muscles differ in their size and physiological characteristics (e.g. fibre type proportion, recruitment strategies, and perfusion capacity) (Enoka, 2008; Zoladz, 2019). Such differences can influence the muscles’ torque production capacity, NMF development and exercise tolerance (*T*_LIM_) (Allen et al., 2008; Gandevia, 2001).

Several studies have compared NMF development across muscle groups. For instance, Vernillo et al. (2018) observed that KE muscles showed a greater decline in maximal voluntary isometric contraction (MVIC) and voluntary activation (VA) compared to the elbow flexors during a 2‐min MVIC, whereas elbow flexors showed greater peripheral fatigue. In contrast, Neyroud et al. (2013) observed a similar decline in MVIC and VA between upper‐ and lower‐limb muscles during submaximal exercise (50% MVIC), whereas peripheral fatigue was more pronounced in upper‐limb muscles. Even considering that muscles performed the task at the same relative intensity to the MVIC, muscle contractions could have been performed at different domains or ranges within the same domain, which can significantly impact NMF and *T*_LIM_. In line with this, a previous study showed that the relative intensity corresponding to the CT of plantar flexors (∼50% MVIC) was greater than for KE muscles (∼29% MVIC), which emphasizes that, even at the same relative intensity to the MVIC, muscles can be stressed in different ways (Abdalla et al., 2018). During the same task in the severe‐intensity domain, the plantar flexors muscles showed a shorter *T*_LIM_ compared to the KE muscles, a finding explained by a smaller impulse above CT (ICT), which represents the finite capacity to sustain exercise above CT (Abdalla et al., 2018). In this context, muscle size likely influences ICT magnitude, although greater active muscle mass may accelerate ICT depletion due to higher metabolic demand, along with other physiological factors. Interestingly, Broxterman et al. (2015) demonstrated that ICT may be determined by the magnitude of NMF accumulated during severe‐intensity exercise. Consistently, our group simultaneously observed that ICT, *T*_LIM_ and peripheral fatigue were increased after 3 weeks of strength training, further emphasizing the link between ICT and NMF outcomes (de Menezes Bassan et al., 2019).

The current study aimed to investigate the effects of severe‐intensity exercise on NMF development, as well as its influence on the loss of torque complexity of the KE and HG muscles in untrained subjects. It was hypothesized that: (1) KE muscles would have a greater *T*_LIM_ and NMF than HG muscles, and (2) NMF would reduce the complexity of torque output for both muscles, with greater impairments for KE than HG muscles.

## 2 METHODS

### 2.1 Ethical approval

This study was approved by the São Paulo State University Institutional Ethics Committee (CAAE 59273422.0.0000.5465). All procedures were conducted according to the *Declaration of Helsinki* on research involving human subjects. All subjects provided written informed consent after receiving a detailed explanation of the procedures before the start of the experiment.

### 2.2 Subjects

Eleven untrained male subjects (23 ± 4 years, 76.2 ± 13.9 kg, 177 ± 6 cm) participated in this study. None of the participants had previous musculoskeletal injuries. The participants were asked to avoid strenuous activity for 24 h prior to exercise tests.

### 2.3 Experimental procedures

**FIGURE 1:**
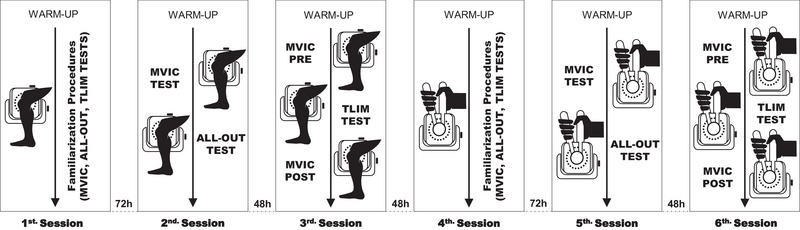
Study design. *Note*: The *T*_LIM_ test is a constant‐load intermittent test at the severe‐intensity domain until task failure. MVIC, maximal voluntary isometric contraction; *T*_LIM_, the limit of tolerance.

All experimental sessions were conducted in a climate‐controlled laboratory (21–23°C) at the same time of the day. All subjects attended the laboratory on six occasions separated by at least 48 h. The first (KE) and fourth (HG) visits were aimed at familiarizing participants with experimental procedures and strength assessments. During the second (KE) and fifth (HG) visits, a 5‐min all‐out test was performed to determine the CT of both muscles. On the third (KE) and sixth (HG) visits, an intermittent constant‐load isometric exercise was performed in the severe‐intensity domain until task failure. Figure 1 shows an overview of the study design.

### 2.4 Dynamometer settings

All tests were conducted on an isokinetic dynamometer (Biodex System 3, Biodex Medical Systems, Shirley, NY, USA). For KE tests, subjects were positioned in a sitting posture on the dynamometer seat with their hip and knee joints at 85° and 75° (0° = full knee extension), respectively. The axis of the dynamometer load cell was aligned with the knee flexion–extension axis, and the lever arm was attached to the subject's shank with a strap (de Menezes Bassan et al., 2019). For HG tests, subjects were positioned in a sitting posture on the dynamometer seat with their shoulder joints flexed at 90° and their dominant arm supported by an arm attachment. The arm was aligned parallel with the prehension grip attachment (Biodex Work‐Simulation Tools), maintaining a 0° abduction. This position ensured that the fixed lever was aligned with the proximal region of the second to fifth metacarpals and the movable lever was in the proximal region of the second to fifth phalanges of the hand. A bandage was applied to the hand to keep it in the same position throughout tests (Junior et al., 2024).

### 2.5 MVIC

MVIC was performed to assess KE and HG maximal strength. Warm‐ups included 5 min of leg cycling (KE) or 3 min of arm cycling on ergometers (HG), followed by 10 submaximal isometric contractions. MVIC involved three maximal isometric contractions (3 s effort, 2 min rest), using the twitch interpolation technique to evaluate neuromuscular function (Millet et al., 2011). Subjects were instructed to start the contraction as strongly and fast as possible, with verbal encouragement for maximal effort (Burnley, 2009). MVIC corresponded to the peak torque attained during MVIC trials.

### 2.6 Parameters of the critical torque model

The CT and ICT of the KE and HG muscles were estimated using Burnley's (2009) method. The 5‐min all‐out test consisted of 60‐MVIC (3 s effort, 2 s rest). Subjects were informed about their MVIC and instructed to reach or pass it during the first three contractions of the test, and continuously encouraged throughout the test, without information about the elapsed time or contractions to avoid any pacing strategy. The CT corresponded to the mean torque of the final six contractions, and ICT was calculated as the sum of impulses above CT from each contraction using the area under the torque × time curve (Burnley, 2009).

### 2.7 T_LIM_

A constant‐load isometric intermittent test (3 s effort, 2 s rest) was performed at an intensity 15% above the CT (i.e. 115% CT) until task failure to evaluate *T*_LIM_. Subjects were instructed to start the contraction quickly, maintaining the target torque, resulting in a rectangular‐shaped contraction. Task failure corresponded to the first of three consecutive contractions where subjects could not reach and maintain the target torque despite verbal encouragement. To evaluate the rate at which NMF develops, MVICs were performed at the task beginning, every 1 min, and at task failure.

### 2.8 NMF assessment

Contractions elicited by transcutaneous electrical stimulation were induced using a high‐voltage constant‐current stimulator (DS7AH, Digitimer, Welwyn Garden City, UK) with a 200‐µs square‐wave stimulus. For femoral nerve stimulation, the cathode (0.5 cm diameter, Dermatrode, American Imex Irvine, CA, USA) was placed over the femoral triangle, with the anode (5 × 10 cm; Compex, Ecublens, Switzerland) positioned on the gluteal fold opposite the cathode (Burnley, 2009). For median nerve stimulation, the anode (0.5 cm diameter, Dermatrode) was positioned distal to the olecranon process, while the cathode was placed over the median nerve in the anterior region of the forearm (Junior et al., 2024). An incremental electrostimulation protocol, which started at 100 mA and increased by 25 mA until torque reached a plateau, was used to determine maximal femoral and median nerve stimulation, followed by a 30% increase to ensure supramaximal stimulation (Hammer et al., 2020). Neuromuscular function was assessed using twitch interpolation, applying supramaximal electrostimulation during the MVIC plateau for superimposed and at rest. Decreases in resting twitch and VA were considered indicating peripheral and central fatigue, respectively. Global fatigue was assessed by the difference in MVIC between the task beginning and task failure. The VA was estimated using the following equations: (1) when superimposed twitch occurs at the peak of MVIC (Burnley, 2009), and (2) corrected for when superimposed twitch did not occur at the peak of MVIC (Broxterman et al., 2015; Hammer et al., 2020; Junior et al., 2024).

(1) $$\mathrm{VA}=\left(1-\frac{\mathrm{Superimposed}\;\mathrm{Twitch}}{\mathrm{Resting}\;\mathrm{Twitch}}\right)\times 100$$

(2) $$\begin{array}{ccc} \mathrm{VA} & = & \left[1-\left(\frac{\mathrm{Torque}\;\mathrm{before}\;\;\mathrm{Superimposed}\;\mathrm{Twitch}\;}{\mathrm{MVIC}}\right)\right.\\ & & \left.\left(\frac{\mathrm{Superimposed}\;\;\mathrm{Twitch}\;}{\mathrm{Resting}\;\mathrm{Twitch}}\right)\right]\times 100 \end{array}$$

where MVIC is a maximal voluntary contraction, and VA is the voluntary activation.

### 2.9 Electromyography

Electromyography was acquired using bipolar Ag–AgCl surface electrodes. The skin was shaved, abraded and cleansed with alcohol before electrode placement on the skin over the vastus lateralis and flexor digitorum superficialis muscles, 2 cm apart (Criswell & Cram, 2011). A reference electrode was placed on the skin over the patella. The starting signal was kept below 5 µV. Electromyography signals were recorded using Miotool (Miotec, Porto Alegre, Brazil), amplified by 10× (total gain of 1000×), and digitalized via an A/D board with a ±5 V input range and 14‐bit resolution. Miograph software (Miotec) acquired the signals at a sampling frequency of 2000 Hz. Electromyography signals were band‐pass filtered (20–500 Hz) using a fourth‐order zero‐lag Butterworth filter over a 1‐s period (i.e. encompassing 0.5 s before and 0.5 s after peak torque). The fatigue index was assessed using root mean square (RMS) and median frequency (MDF) analysis (Merletti et al., 2005). RMS data from submaximal contractions throughout the *T*_LIM_ test were normalized to their baseline MVIC (i.e. RMS_max_). Fatigue‐related changes in muscle activation behaviours were assessed by computing the differences in the RMS and MDF between the first and last 30 s.

### 2.10 Torque analyses

Torque signals were acquired using an isokinetic dynamometer (Biodex System 3) synchronized with a electromyograph (Miotool) and calibrated according to the manufacturer's instructions. Torque signals were sampled at 2000 Hz and analysed using custom‐made scripts written in Matlab R2023a (MathWorks Inc., Natick, MA, USA) (Winter, 2009).

### 2.11 Complexity and magnitude

Raw data for each submaximal contraction of the first and last minutes were used to calculate the SD, CV, ApEn and DFA‐α by acquiring a 2‐s steady contraction period. SD and CV were used to assess absolute variability and variability relative to the mean, respectively (Pethick et al., 2021). ApEn was used to assess regularity or randomness, while DFA‐α was used to assess the temporal fractal scaling of the torque output (Pethick et al., 2021). The ApEn and DFA‐α were calculated based on the earlier studies of Pethick et al. (2016) and Pethick, Winter et al. (2019), using the proposed methods. ApEn quantifies (Equation 3) the negative natural logarithm of the conditional probability that a model of length *m* (defined at 2) is repeated over time (Pincus, 1991; Slifkin & Newell, 1999). Corresponding models that remain arbitrarily similar (within the tolerance, *r*, defined at 10% of SD) are counted, with the number of matches to the *i*th model of length *m* designated *B*_i_. The number of these matches that remain similar to them + 1th point is then counted, with this number for the *i*th model designated *A*_i_. The conditional probability that the model includes the (*m* + 1)th data point, given the model of length *m*, is then calculated for each model match. The negative logarithm of the condition probability is calculated for all models, and the results are averaged. Low values, close to 0, indicate high regularity and low complexity, while high values, close to 2, indicate low regularity and high complexity (Pincus, 1991; Slifkin & Newell, 1999).

(3) $$\mathrm{ApEn}\left(m,r,N\right)=\frac{1}{N-m}\sum_{i=0}^{N-m}.\log\;\frac{A_{i}}{B_{i}}$$

where *N* is the number of data points in the time series, *m* is the length of the model, *A_i_* is the number of matches to the *i*th model of length *m* + 1 data points, and *B_i_* is the number of matches to the *i*th model of length *m* data points.

To perform DFA‐α analysis, the time series is first integrated, and the vertical characteristic scale of this integrated time series is measured (Peng et al., 1994). The integrated time series is then divided into boxes of length *n* and a least‐squares line fitted, representing the trend for each box. The *y*‐coordinate of the straight‐line segment of length *n* in the *k*th box is denoted by *y_n_* (*k*), and the integrated time‐series is detrended by subtracting the local trend in each box (Peng et al., 2002; Stanley et al., 1999). For a given box size, n, the characteristic size of fluctuation for the integrated and detrended time‐series is based on equation 4.

(4) $$F\left(n\right)=\sqrt{\frac{1}{N}\sum_{k=1}^{N}.\left[y\left(k\right)-Y_{n}\left(k\right)\right]2}$$

This procedure is repeated over all time scales of box sizes to provide a relationship between box size and *F*(*n*). Based on the methods of Pethick et al. (2016) and Pethick, Winter et al. (2019), 57 boxes, ranging from 1250 to 4 data points, were used for analysis. The slope of the log–log plot of *n* and *F*(*n*) determines the scaling parameter, α. When α = 0.5, the time series is completely random, that is, white noise, and completely independent of the previous values. When 0.5 < α < 1, power law correlations are observed, while for α > 1, there are correlations, but it does not take the form of a power law. α = 1 is indicative of pink noise, while α = 1.5 is indicative of Brownian noise. In these conditions, when α ≠ 0.5, the values are not completely independent and are correlated with the previous values (Pethick et al., 2021).

### 2.12 Statistical analyses

The Shapiro–Wilk test was used to assess normality, as normality was not confirmed for most variables, except for ApEn and DFA‐α; therefore, a non‐parametric statistical procedure was applied to analyse the data. The results are presented as means ± SD. Levene's test was used to confirm the homogeneity of variance. Parametric data comparisons were performed using Student's *t*‐test, and non‐parametric data using the Mann–Whitney test. For NMF variables, a one‐way ANOVA with repeated measures was used, while for complexity analyses, a two‐way ANOVA (time × muscle) was applied. When a significant time × muscle interaction was identified, pairwise analyses were performed using one‐way ANOVA for matched (time) and independent samples (muscle group), followed by Bonferroni's *post‐hoc* test. The significance level was set at *P < *0.05. Data were analysed using a statistical software package (SPSS Version 20.0; IBM Corp., Armonk, NY, USA).

## 3 RESULTS

### 3.1 *T*_LIM_ to severe‐intensity exercise

In absolute comparisons, KE muscles showed greater CT (KE 76.5 ± 16.0 N·m × HG 25.0 ± 5.63 N·m, *P*< 0.001) and ICT (KE 7225.1 ± 2405.9 N·m·s × HG 1583.2 ± 639.7 N·m·s, *P* < 0.001) compared to HG muscles. In contrast, there were no differences in the CT relative to isometric peak torque (KE 32.8 ± 7.39% × HG 33.8 ± 7.87%, *P* = 0.818), and there were no significant differences in the mean torque performed above CT (KE 14.6 ± 3.10% × HG 14.8 ± 4.14%, *P* = 0.847) during the constant‐load isometric intermittent test. Similarly, the time to task failure during the constant‐load isometric intermittent test did not differ between KE and HG muscles (KE 412.9 ± 178.8 s × HG 319.0 ± 89.1 s, *P* = 0.270).

### 3.2 Electromyography, aetiology and the rate of NMF development

There was a significant time effect in the MVIC (*F*_(KE)_ = 48.7, *P* < 0.001; *F*_(HG)_ = 75.9, *P* < 0.001), resting twitch (*F*_(KE)_ = 67.3, *P* < 0.001; *F*_(HG)_ = 31.9, *P* < 0.001), and VA (*F*_(KE)_ = 15.9, *P* < 0.001; *F*_(HG)_ = 23.0, *P* = 0.003) for both muscles. However, in relative comparisons, KE muscles showed a greater decrease in VA (*P* < 0.001) and resting twitch (*P *< 0.001) compared to the HG muscles, but there were no differences in MVIC decrease (*P* = 0.193) between them (Table 1). Similarly, KE muscles exhibited a faster reduction in resting twitch (*P* < 0.001) compared to HG muscles, while there were no differences for VA (*n *= 10) (*P* = 0.863) (Table 1). However, HG muscles showed a faster decrease in MVIC (*P* = 0.023).

**TABLE 1:** NMF and electromyography: effects of severe‐intensity exercise on the KE and HG muscles’ performance.

	KE	HG
MVIC		
MVIC at task beginning (N·m)	236.5 ± 54.1	74.4 ± 16.0
MVIC at task failure (N·m)	136.7 ± 32.3**	39.1 ± 8.48**
ΔMVIC (%)	−40.5 ± 14.4	−46.7 ± 9.62
ΔMVIC/Δ*t* (% min^−1^)	−3.34 ± 1.77	−4.96 ± 2.54*
Resting twitch		
Resting twitch at task beginning (N·m)	50.8 ± 10.1	12.2 ± 1.96
Resting twitch at task failure (N·m)	17.7 ± 9.28**	8.53 ± 1.68**
ΔResting twitch (%)	−63.8 ± 20.9	−29.0 ± 14.5*
ΔResting twitch/Δ*t* (%.min^−1^)	−7.52 ± 3.71	−3.63 ± 1.85*
VA		
VA at task beginning (%)	88.0 ± 6.53	96.3 ± 3.96
VA at task failure (%)	73.1 ± 13.4**	88.9 ± 6.03**
ΔVA (%)	−14.9 ± 12.4	−7.66 ± 5.23*
ΔVA/Δ*t* (%.min^−1^)	−1.15 ± 1.13	−0.95 ± 0.63
RMS		
RMS at task beginning (%RMS_max_)	34.0 ± 10.1	32.1 ± 9.63
RMS at task failure (%RMS_max_)	74.1 ± 24.6**	51.5 ± 22.8**
ΔRMS (%RMS_max_)	40.1 ± 21.0	19.4 ± 20.9*
MDF		
MDF at task beginning (Hz)	92.4 ± 14.2	101.8 ± 14.3
MDF at task failure (Hz)	79.0 ± 12.3**	87.1 ± 18.5**
ΔMDF (Hz)	−13.4 ± 8.98	−14.6 ± 8.46

Values are presented as means ± SD. *Significantly different from the KE muscles; **significantly different from the task beginning. Data for the VA were available for only 10 subjects in the analysis of the rate of NMF development. MDF, median frequency; MVIC, maximal voluntary isometric contraction; RMS, root mean square; VA, voluntary activation.

There was a significant time effect in the RMS (*F*_(KE)_ = 25.6, *P* < 0.001; *F*_(HG)_ = 9.26, *P* = 0.012) and MDF (*F*_(KE)_ = 24.6, *P* = 0.001; *F*_(HG)_ = 33.0, *P* < 0.001) for both muscles. However, KE muscles showed a greater ΔRMS compared to HG muscles (*P* = 0.038). There were no significant differences in ΔMDF (*P* = 0.718) between them (Table 1).

### 3.3 Complexity and magnitude

**FIGURE 2:**
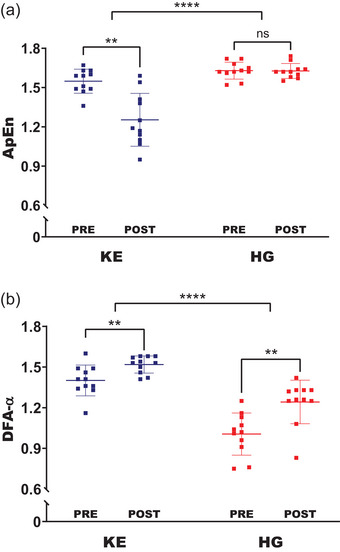
Mean (±SD) changes in muscle groups for ApEn (a) and DFA‐α (b) at task beginning (PRE) and failure (POST) in 11 participants (*n* = 11). Individual values for each subject are also shown. **Significantly different from task beginning (PRE); ****significantly different from KE muscles at both task beginning (PRE) and failure (POST); ns, non‐significant. ApEn, approximate entropy; DFA‐α, detrended fluctuation analysis; HG, handgrip; KE, knee‐extensor.

There was significant time × muscle interaction (*F* = 23.2, *P* < 0.001) for ApEn. *Post hoc* analysis revealed that ApEn decreased over time for KE muscles (*P* < 0.001), while ApEn remained unchanged for HG muscles (*P* = 0.950) at task failure (Table 2). Furthermore, HG muscles showed greater ApEn than KE muscles at the task's beginning (*P* = 0.027) and failure (*P* < 0.001) (Figure 2a). Similarly, there was a significant interaction between time × muscles (*F* = 7.51, *P* = 0.013) for DFA‐α (Table 2). *Post hoc* analysis revealed that DFA‐α increased over time for KE (*P* = 0.001) and HG muscles (*P* < 0.001). However, HG muscles showed lower DFA‐α than KE muscles at task beginning and failure (*P* < 0.001) (Figure 2b). In addition, KE muscles showed a greater ΔApEn than HG muscles (*P* < 0.001), while HG muscles showed a greater Δ DFA‐α than KE muscles (*P* = 0.013) (Table 2). Similarly, ApEn decreased faster (*P* < 0.001), while DFA‐α increased slower (*P* = 0.019) for KE muscles compared to HG muscles (Table 2).

**TABLE 2:** Magnitude and complexity: effects of the severe‐intensity exercise on torque fluctuations of the KE and HG muscles.

	KE	HG
Standard deviation		
SD at task beginning (N·m)	3.04 ± 0.80	0.99 ± 0.39^†^
SD at task failure (N·m)	6.01 ± 2.63**	1.34 ± 0.44^#^
ΔSD (N·m)	2.97 ± 2.52	0.35 ± 0.52*
ΔSD/Δ*t* (N·m.min^−1^)	0.51 ± 0.47	0.08 ± 0.12*
Coefficient of variation		
CV at task beginning (%)	3.71 ± 1.73	3.91 ± 1.64
CV at task failure (%)	8.40 ± 5.63**	5.41 ± 1.81**
ΔCV (%)	4.69 ± 5.74	1.50 ± 2.20
ΔCV/Δ*t* (%.min^−1^)	0.76 ± 0.89	0.34 ± 0.49
Approximate entropy		
ApEn at task beginning	1.55 ± 0.09	1.63 ± 0.06^†^
ApEn at task failure	1.25 ± 0.20**	1.63 ± 0.06^#^
ΔApEn	−0.29 ± 0.20	0.00 ± 0.05*
ΔApEn/Δ*t*	−0.05 ± 0.04	0.00 ± 0.01*
DFA‐α		
DFA‐α at task beginning	1.40 ± 0.11	1.01 ± 0.16^†^
DFA‐α at task failure	1.52 ± 0.06**	1.24 ± 0.16**^, #^
ΔDFA‐α	0.12 ± 0.11	0.24 ± 0.09*
ΔDFA‐α/Δ*t*	0.02 ± 0.02	0.05 ± 0.02*

Values are presented as mean ± SD. *Significantly different from KE muscles; **significantly different from task beginning; †significantly different from KE muscles at the task beginning; #significantly different from KE muscles at task failure. ApEn, approximate entropy; CV, coefficient of variation; DFA‐α, detrended fluctuation analysis; SD, standard deviation.

**FIGURE 3:**
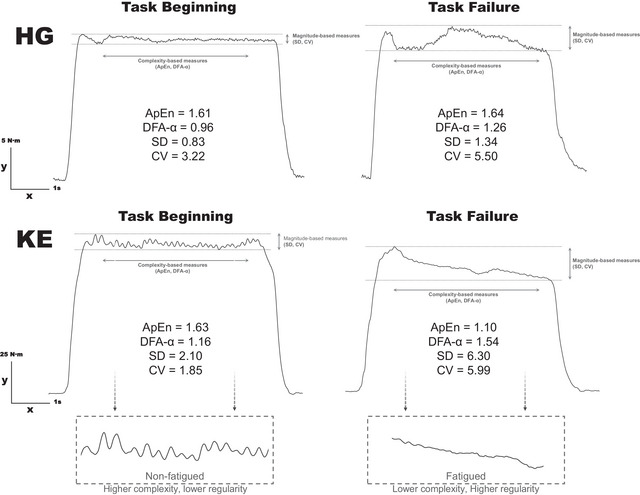
The raw torque output during representative contractions at the beginning and at task failure for knee extensors (KE) and handgrip (HG) muscles. The beginning (first minute of exercise) corresponds to the initial performance of KE and HG. In contrast, task failure (last minute of exercise) is defined as the inability to maintain the target torque for three consecutive contractions. Note that for both muscles, at task failure, a constant rectangular‐shaped contraction is not maintained. Nevertheless, changes in fluctuation responses can be observed across all variables in the KE muscles, whereas this is not the case for HG muscles. Magnitude‐based measures (SD and CV) refer to the amount of variability in the torque output during contraction. Complexity‐based measures refer to the regularity or randomness in the torque output (ApEn) or its temporal fractal scaling behaviour (DFA‐α). Both variability and complexity can be influenced by multiple factors, including neuromuscular characteristics (e.g. number of motor units and their recruitment thresholds); exercise conditions (e.g. low × high intensity); and the onset of neuromuscular fatigue. These factors may contribute to increasing the common synaptic input, commonly evidenced as low‐frequency oscillatory responses. ApEn, approximate entropy; CV, coefficient of variation; DFA‐α, detrended fluctuation analysis; SD, standard deviation.

There was significant time × muscle interaction (*F* = 11.4, *P* = 0.003) for SD. *Post hoc* analysis revealed that SD increased over time for KE muscles (*P* < 0.001), while it remained unchanged for HG muscles (*P* = 0.527). Furthermore, HG muscles showed lower SD than KE muscles at task beginning and failure (*P* < 0.001) (Table 2). In contrast, there was no time × muscle interaction (*F* = 2.96, *P* = 0.101) and muscle effect for CV (*F* = 1.98, *P* = 0.175), only a time effect (*F* = 11.2, *P* = 0.003) (Table 2). In addition, KE muscles showed a greater increase in SD than HG muscles (*P* < 0.001), while CV did not differ between them (*P *= 0.065) (Table 2). Similarly, SD increased faster (*P* = 0.003) for KE muscles than HG muscles, while there were no differences for CV between them (*P* = 0.193) (Table 2). Raw torque output during representative contractions at the beginning and at task failure for both KE and HG muscles is shown in Figure 3.

## 4 DISCUSSION

The findings of this study further support the growing body of evidence that NMF reduces torque complexity. During severe‐intensity exercise, it has been shown that NMF reduced torque complexity in KE and HG muscles. However, in HG muscles, this was observed only by DFA‐α analysis. In addition, loss of torque complexity was accompanied by greater peripheral and central fatigue in the KE muscles compared to the HG muscles. In applied terms, this decline in complexity usually reflects an impaired neuromuscular system's adaptability in responding to external perturbations, which leads to impacts on muscle torque control (Pethick et al., 2021). Although a wide range of studies have been conducted using the same muscle group, our novel findings suggest that the KE and HG muscles exhibited different levels of loss of torque complexity.

In line with the findings of Pethick et al. (2015, 2016), Pethick, Winter et al. (2019) and Pethick, Whiteaway et al. (2019), as NMF develops, impaired central and peripheral mechanisms contribute to reduce the ability of skeletal muscles to produce torque, as well as to control it (Figure 3). Markers of the NMF aetiology showed that KE muscles experienced greater central and peripheral fatigue than HG muscles. However, muscle torque‐generating capacity declined by a similar magnitude between them. Notably, despite differences in ICT, both muscles exhibited a similar *T*_LIM_ at the same task. In contrast, although both muscles showed significant NMF effects, only the KE muscles experienced reductions in ApEn at task failure. Similarly, Pethick et al. (2016) have shown that KE muscles experienced a significant loss of torque complexity (i.e. a decrease in ApEn) during severe‐intensity exercise. However, some aspects should be considered when interpreting the findings for the HG muscles. The lack of effect in ApEn does not mean that torque complexity remained preserved; rather, it may reflect limitations of ApEn, which alone does not detect such changes. For this reason, additional measurements are recommended to characterize adequately the loss of torque complexity (Pethick et al., 2021).

In this context, a more comprehensive investigation of torque fluctuation patterns is required, considering their complexity (i.e. DFA‐α or even sample entropy and multiscale entropy) or variability (Pethick et al., 2021). Variability can be assessed using traditional approaches such as SD and CV of torque output, as well as using newer approaches like force unsteadiness (for further details, see Yacoubi & Christou, 2024). It has been shown that fluctuations in torque production are influenced by multiple factors that, over time, reduce their accuracy (Lodha & Christou, 2017). For instance, increased exercise demands require greater neural drive, thus reducing their control capacity. Notably, this decline is associated with increased low‐frequency oscillation responses (Lodha & Christou, 2017). Furthermore, neuromuscular characteristics (e.g. number of motor units and their recruitment threshold) seem to be key determinants of the magnitude of oscillation in the neural drive (Enoka & Farina, 2021). Similarly, NMF development, characterized by a progressive reduction in the contractile capacity, further contributes to the fluctuation's enhancement and reduced torque control capacity (Castronovo et al., 2015). Although these factors may differ between different muscles, both HG and KE muscles performed the exercise to task failure under similar metabolic demand and demonstrated comparable CV responses at task beginning, which suggests minimal impacts on the subsequent analysis of NMF effects between these muscle groups.

To our knowledge, our findings are the first to demonstrate that NMF induces a loss of torque complexity in two different muscles (KE and HG muscles). Notably, in HG muscles, loss of torque complexity was detected only through DFA‐α analysis, while ApEn remained unchanged. An explanation for this may be related to the different sensitivity of DFA‐α and ApEn in assessing torque complexity, thus highlighting the different aspects of their dynamics (Pincus, 1991; Slifkin & Newell, 1999). As DFA‐α is sensitive to long‐range correlations, it can detect different intrinsic properties of the system because of the analyses performed on different scales, whereas ApEn measures predictability within a time series, which may not detect changes if torque regularity remains unchanged (Pethick et al., 2021; Pincus, 1991; Slifkin & Newell, 1999). These findings suggest that even with a significant NMF effect, HG muscles showed an unchanged torque regularity (ApEn). However, its fractal dynamics were more predictable and less complex at task failure.

In addition, it is noteworthy that HG muscles showed lower DFA‐α but greater ApEn at the task beginning and failure, respectively, compared to KE muscles. However, KE muscles showed a greater ΔApEn compared to HG muscles from task beginning to task failure, whereas ΔDFA‐α was greater for HG muscles. Although DFA‐α was more affected in the HG muscles, it remained at lower α values (i.e. demonstrating that KE and HG muscles were in different noise ranges). In this sense, HG muscles started and completed the severe‐intensity exercise within the pink noise range (1/*f*) (i.e. classified as an optical noise to the system), while KE muscles started within the pink noise range but transitioned to the Brownian noise range by the end of the exercise (Sejdić & Lipsitz, 2013). Within this range, noise overlaps with the signal, demonstrating that at task failure, KE muscles showed a reduced neuromuscular adaptability, even more so than HG muscles (Sejdić & Lipsitz, 2013). These changes occurred alongside the development of greater and faster peripheral fatigue. In contrast, central fatigue impairment was greater for KE muscles only at task failure, despite a similar MVIC at task failure between muscles, with a faster decline observed in HG muscles. Similarly, KE muscles showed a greater increase in RMS, while MDF remained similar between them. Therefore, the greater loss of torque complexity exhibited by KE muscles could be related to a greater central and peripheral fatigue impairment, which led to progressively increasing requirements for additional motor unit recruitment or increased firing rate during severe‐intensity exercise, as suggested by the greater ΔRMS (Merletti et al., 2005).

In a previous study, Castronovo et al. (2015) demonstrated that as NMF develops, there is an increased common synaptic input, as well as an increased torque fluctuation. In fact, the common synaptic input has been considered the major determinant of torque variability (SD and CV) (Farina & Negro, 2015). Based on the different KE and HG muscle size, a greater SD would be expected for KE muscles than HG muscles at the beginning and at task failure, because of their necessity to recruit and sustain the activity of larger motor units (Enoka, 2008). Accordingly, our findings showed that the KE muscles exhibited greater SD than HG muscles at the beginning and at task failure, but when torque variability is normalized to the mean (i.e. CV), there were no significant differences. NMF was responsible for significantly increasing SD during the severe‐intensity exercise for KE muscles; however, even in the presence of a significant NMF effect, SD remained unchanged for HG muscles. In addition, KE showed a greater ΔSD compared to HG muscles from task beginning to task failure. Consistent with this, Pethick et al. (2015, 2016, 2020), Pethick, Winter et al. (2019) and Pethick, Whiteaway et al. (2019) have shown that the torque variability of KE muscles is affected by NMF under several conditions. Although the effects of NMF on torque variability in HG muscles during severe‐intensity exercise are poorly understood, our findings suggest that the unchanged SD at task failure may reflect their lower susceptibility to central and peripheral fatigue. This may also be indicated by a lower requirement for additional motor unit recruitment or increased firing rate, as evidenced by the lower ΔRMS (Merletti et al., 2005). However, NMF similarly affected the CV of both KE and HG muscles, with no differences between them. The difference in CV between KE and HG muscles approached significance (*P* = 0.065), which limits the findings’ conclusion and may be attributed to the small sample size (*n* = 11) and high variation in this measurement. Taken together, these findings demonstrated that, at the same task within the severe‐intensity domain, NMF induces a consistent increase in torque variability for the KE muscles, but with caveats for the HG muscles, as well as possible differences between muscles, due either to size or NMF differences.

### 4.1 Limitations

Some limitations should be considered when interpreting these findings. Even though to quantify torque complexity a relatively short time series is required, longer contraction periods could be more appropriate, allowing for a prolonged steadiness profile during contractions. However, previous studies have typically employed many contraction duty cycles, particularly for HG muscles, and Broxterman et al. (2014) demonstrated that, during dynamic HG exercise, a longer contraction duty cycle can impair blood flow and O_2_ extraction, resulting in shorter *T*_LIM_. Similarly, it changes muscle activity, as observed in RMS and MDF outcomes, which can ultimately lead to different muscular recruitment strategies. Thus, during the pilot study, we observed that a mid‐range contraction duty cycle (i.e. 3 s of effort, 2 s of rest) would be necessary to allow an equal comparison between the KE and HG muscles. To correct potential errors in VA estimation, different equations were adopted, each accounting for different factors, such as the torque at which stimulation was applied relative to the MVIC during the contraction. Nevertheless, using these approaches together may introduce some inaccuracies. Another limitation was the lack of adequate control of caffeine consumption, which could influence *T*_LIM_ and NMF outcomes. Furthermore, this study did not include direct measurements of motor unit characteristics, muscle perfusion, architectural characteristics, or fibre‐type composition, which limits the ability to identify the mechanisms underlying the loss of torque complexity between different muscle groups.

### 4.2 Conclusions

During severe‐intensity exercise, NMF reduced torque complexity in the KE and HG muscles, with greater impairments occurring for the KE muscles. The greater loss of torque complexity exhibited by KE muscles was accompanied by greater central and peripheral fatigue compared to HG muscles, despite a similar decline in MVIC and *T*_LIM_ at task failure. Therefore, the magnitude of NMF may influence the loss of torque complexity between different muscles, resulting in greater fluctuations in torque output and consequently impaired motor control ability. Furthermore, the greater impairment observed may be influenced by differences in muscle size, which could affect various physiological responses.

## AUTHOR CONTRIBUTIONS

Rubens C. Junior, Renan V. Barreto, Benedito S. Denadai, and Camila C. Greco designed this research. Rubens C. Junior, Renan V. Barreto, Gabriel Fontanetti, and Leonardo C.R. de Lima performed the experiment. Rubens C. Junior, Renan V. Barreto, and Camila C. Greco analysed and interpreted the results. Rubens C. Junior prepared figures. Rubens C. Junior and Camila C. Greco drafted the manuscript. Rubens C. Junior, Renan V. Barreto, Gabriel Fontanetti, Leonardo C.R. de Lima, Benedito S. Denadai, and Camila C. Greco edited and revised the final version of the manuscript. All authors have read and approved the final version of the manuscript and agree to be accountable for all aspects of the work in ensuring that questions related to the accuracy or integrity of any part of the work are appropriately investigated and resolved. All persons designated as authors qualify for authorship, and all those who qualify for authorship are listed.

## CONFLICT OF INTEREST

No potential conflict of interest is reported by the authors.

## Data Availability

Data are available on reasonable request.
